# Does a narcissism epidemic exist in modern western societies? Comparing narcissism and self-esteem in East and West Germany

**DOI:** 10.1371/journal.pone.0188287

**Published:** 2018-01-24

**Authors:** Aline Vater, Steffen Moritz, Stefan Roepke

**Affiliations:** 1 Freie Universität Berlin, Department of Education and Psychology, Berlin, Germany; 2 University of Potsdam, Department of Psychology, Potsdam, Germany; 3 Charité–Universitätsmedizin Berlin, Campus Benjamin Franklin, Department of Psychiatry and Psychotherapy, Berlin, Germany; 4 University Medical Center Hamburg-Eppendorf, Department for Psychiatry and Psychotherapy, Hamburg, Germany; National Cheng Kung University, TAIWAN

## Abstract

Narcissism scores are higher in individualistic cultures compared with more collectivistic cultures. However, the impact of sociocultural factors on narcissism and self-esteem has not been well described. Germany was formerly divided into two different social systems, each with distinct economic, political and national cultures, and was reunified in 1989/90. Between 1949 and 1989/90, West Germany had an individualistic culture, whereas East Germany had a more collectivistic culture. The German reunification provides an exceptional opportunity to investigate the impact of sociocultural and generational differences on narcissism and self-esteem. In this study, we used an anonymous online survey to assess grandiose narcissism with the Narcissistic Personality Inventory (NPI) and the Pathological Narcissism Inventory (PNI) to assess grandiose and vulnerable aspects of narcissism, and self-esteem with the Rosenberg Self-Esteem Scale (RSE) in 1,025 German individuals. Data were analyzed according to age and place of birth. Our results showed that grandiose narcissism was higher and self-esteem was lower in individuals who grew up in former West Germany compared with former East Germany. Further analyses indicated no significant differences in grandiose narcissism, vulnerable narcissism or self-esteem in individuals that entered school after the German reunification (≤ 5 years of age in 1989). In the middle age cohort (6–18 years of age in 1989), significant differences in vulnerable narcissism, grandiose narcissism and self-esteem were observed. In the oldest age cohort (> 19 years of age in 1989), significant differences were only found in one of the two scales assessing grandiose narcissism (NPI). Our data provides empirical evidence that sociocultural factors are associated with differences in narcissism and self-esteem.

## Introduction

Are modern capitalistic cultures nurturing narcissism? Sociocultural changes are frequently proposed to be central mechanisms contributing to increasing narcissism [1]. The majority of empirical studies on the associations between sociocultural changes and narcissism relied on student samples from the US. In the present study, we examined an exceptional case in human history: The division and reunification of Germany. We investigated whether individuals exposed to these two different social systems (with distinct economic, political and national cultures) differed in grandiose and vulnerable narcissism, and self-esteem.

### Empirical evidence for the narcissism epidemic

Narcissism is increasing in modern Western societies and this has been referred to as a “narcissism epidemic” [1]. The endorsement rate for the statement “I am an important person” has increased from 12% in 1963 to 77–80% in 1992 in adolescents [2]. Recently published books feature more self-centered language compared with earlier publications. For instance, the personal pronouns *I* and *me* are used more frequently than *we* and *us* [3]. Moreover, the use of narcissistic phrases such as “I am the greatest” has increased between 1960 and 2008 [4]. The rise of narcissism is also reflected in more self-focused song lyrics [5] and a stronger orientation towards fame in TV shows [6]. These observations suggest that narcissistic expressions within individualistic cultures have become more frequent.

Scores of self-reported grandiose narcissism, assessed by the Narcissistic Personality Inventory (NPI), have increased [7, 8]. Twenge and Campbell reported a significant increase in NPI scores in a cross-temporal meta-analysis of American college students between 1979 and 2006 [9]. NPI scores were 30% higher in the most recent cohort compared with the first cohort. Further analyses between 2002 and 2007 excluded any confounding effects of ethnicity. Taken together with other studies, these findings confirmed that NPI scores are increasing over time in American college students ([10], but also see [13]).

Evidence for an increase in narcissism in Western societies has predominantly been provided by the same research group. In these studies, narcissism was consistently measured using the NPI, which has received much criticism [10–12]. Shortcomings of the NPI include a restriction to grandiose aspects of narcissism [13] and problems with validity [14]. Moreover, the NPI is constructed based on the clinical definition of Narcissistic Personality Disorder (NPD) in the third edition of the Diagnostic and Statistical Manual of Mental Disorders (DSM) produced by the American Psychiatric Association [15]. Nevertheless, Raskin and Terry developed the NPI to assess individual differences in nonclinical populations. The authors have themselves admitted that the NPI fails to capture important psychological and behavioral dimensions that are inherent to pathological narcissism [7]. This means that high NPI scores may represent a non-distressed, self-confident version of grandiose narcissism [13]. However, recent research shows that the NPI provided a strong match to expert ratings of DSM-IV-TR NPD and grandiose narcissism, compared to other narcissism inventories [16, 17].

The Pathological Narcissism Inventory (PNI) has been developed to capture both grandiose and vulnerable aspects of narcissism [13]. PNI scores are thought to be closely related to the clinical diagnosis of NPD, although this has not yet been tested in narcissistic patients. However, recent evidence indicates that the PNI is a useful tool for assessing more pathological aspects of narcissism [18].

Self-esteem, defined as global evaluation of the self [19], is related to narcissism [14, 20–22]. However, recent data provide evidence that narcissism differs from self-esteem in various domains, such as its phenotype, its consequences, its development, and its origins [23]. Self-esteem and narcissism have distinct impacts on outcome measures [21, 24–28]. Narcissism and high self-esteem both include positive self-evaluations, but the entitlement, exploitation, sense of superiority, and negative evaluation of others that are associated with narcissism are not necessarily observed in individuals with high self-esteem [29]. Self-esteem is increasing in the Western world. Middle school students from the United States had markedly higher self-esteem scores in the mid-2000s compared with the late 1980s [30]. Moreover, the self-esteem of college students increased substantially between 1968 and 1994 [31].

### Sociocultural environments and narcissism

The development of personality traits is closely related to the cultural environment [32–35]. Cultural environments can be classified as individualistic or collectivistic [36, 37]. Individualistic cultures encourage a stronger focus on the self, whereas collectivistic cultures emphasize the importance of social values. Narcissism contains a strong focus on the self, accompanied by a high need for admiration, and grandiose fantasies [38, 39]; therefore members of individualistic cultures may be more narcissistic than individuals from collectivistic cultures [40].

Some studies have provided empirical evidence that individualistic values can lead to narcissism [41]. Individuals from the United States, which has a more individualistic culture, have higher grandiose narcissism scores (NPI) compared with individuals from Asian countries and the Middle East, which have a more collectivistic culture [40]. Likewise, Chinese people have lower self-enhancement scores than individuals from more individualistic Western societies, such as the United States, United Kingdom, Canada, Australia, and Europe [42].

Lower self-esteem has been reported in individuals from East Asia than Western cultures [43]. Similarly, Japanese college students had lower self-esteem scores than Westerners [44–47]. Lower self-esteem in collectivistic societies may be due to the fact that importance is not focused on the self but rather given to the group. Taken together, these findings suggest that individual differences in self-esteem and narcissism may be explained by distinguishable characteristics inherent to different cultures.

### The effect of age on narcissism

Past research indicates that narcissism decreases with age. Younger individuals have higher narcissism scores [48–50] than older individuals. Wilson and Sibley identified a curvilinear effect of age on NPI scores in the general New Zealand population [51]; 9.4% of individuals in their twenties reported symptoms of NPD, compared with 3.2% of individuals older than 65 years. Hypersensitive narcissism is a variant of pathological narcissism and can be measured by the Hypersensitive Narcissism Scale [52]. Scores using this scale correlated negatively with age [53].

Empirical research provides evidence that entering adulthood during a recession tempers later narcissism, i.e., participants who come of age during tumultuous economic times are less likely to endorse narcissism relative to people who enter adulthood in more prosperous times [54, 55]. Bianchi argues that economic conditions shape later attitudes and values that reflect the prevailing concerns of the time. Narcissistic self-focus may be viable only when people are not reliant on others to satisfy their basic needs during economic prosperous times [56, 57]. In line with this, in Finnish 18 year old man born between 1962 and 1976 a steady increase in personality traits that are associated with higher earnings in later life (e.g., self-confidence, sociability, leadership motivation) has been observed [58].

Self-esteem also changes with age. Latent growth curve analyses have revealed that self-esteem increases with age, peaks at 60 years, and then declines into old age (≥ 70 years) [59–61]. According to Orth and colleagues, individuals establish their sense of self, including their roles, values, and interests during adolescence and young adulthood [59]. Self-esteem increases with age as personality traits mature and social roles begin to manifest. It then decreases during old age because individual traits (e.g., physical strength) and social surroundings (e.g., empty nest syndrome, loss of partner) begin to change. Another study shows that birth cohort has a measurable influence on self-esteem through its interaction with age. Participants born in later years (e.g., 1960) score higher in self-esteem than participants born in earlier years (e.g., 1920) [62]. Moreover, the authors compared participants of the same age in 1986 versus 2002 from the Americans’ Changing Lives (ACL) cohort-sequential study. According to their results, participants of the same age in 2002 scored higher in self-esteem than those in 1986. The authors argue that cultural change in the form of cohort and time period have to be considered when assessing self-esteem in cross-sectional and longitudinal studies.

### Rationale of the present study

Cultural transformation towards more individualistic values in Western societies has been blamed for the rise in narcissism [34, 63]. However, evidence for an increase in narcissism has largely come from NPI scores of college student cohorts collected between the late 1970s and 2010 [63]. Many researchers have argued that this is insufficient evidence for a narcissism epidemic in Western societies [64, 65]. Moreover, the NPI has been criticized as a measure of narcissism [13, 14]. To address these concerns, we have included evaluation by the PNI in our study, which assesses grandiose *and* vulnerable narcissism and complements information from the NPI [18].

To date, the influence of culture on narcissism has been investigated using cross-temporal birth cohorts [9, 66] or by comparing samples from different cultural environments [40, 42, 67]. These approaches have methodological limitations. Cross-temporal cohorts use the same self-reporting method and can be affected by sampling biases. Trzesniewski and Donnellan reported that population-based inferences cannot be made from cross-temporal meta-analytic studies that are based on convenience sampling [65]. There are also problems with the use of trans-cultural cohorts, including differences in language use and problems with the translation of self-reporting questionnaires used in these studies. Culture is transmitted through language [68]; therefore it is impossible to rule out mediating variables in language use and word meaning between cohorts from different cultures.

To address the methodological restrictions of cross-temporal birth cohorts and cross-cultural comparisons, we used a historically exceptional research setting: reunified Germany. Germany shares the same national culture, but was divided into two independent societies and political systems after World War II. In 1949, the German Democratic Republic (GDR, or East Germany), was founded on the territory of the Russian sector as a communist society with a collectivistic ideology. In the same year, the Federal Republic of Germany (FRG, or West Germany) was founded as capitalist society on the territory of the Trizone (including the American, British, and French sector). Between 1961 and 1989, interchange between these two German regions was heavily restricted. The Berlin Wall fell in November 1989, marking the beginning of unification. In October 1990, Germany finally became a unified, capitalism-orientated society.

The reunification of the socialist GDR and the democratic FRG after more than four decades of separation can be considered a “natural experiment” [69–71]. Until 1945, East and West Germany shared central cultural elements, including language, common norms, values, and habits. After 1945, East Germany gradually became a communist society and its people were increasingly exposed to collectivism. In contrast, West Germany retained its capitalistic system and developed a more individualistic culture [72, 73]. As a result, the German population now consists of a younger generation that grew up under the individualistic system and an older population that were raised either in collectivistic East Germany or individualistic West Germany. Comparing individuals from these two geographical regions may reveal to what degree cultural conditions shape personality across age cohorts.

### Aims and hypotheses

#### Main effect for group

The aim of the current study was to compare grandiose narcissism, vulnerable narcissism and self-esteem between individuals who grew up in former East and West Germany. First, based on previous findings [40, 42], we hypothesized that grandiose narcissism would be more prevalent in individuals from West Germany, who have been exposed to individualistic views compared with individuals from collectivistic East Germany.

Second, we aimed to determine whether individuals from East and West Germany show differences in vulnerable narcissism. Since narcissistic vulnerability in different cultures has not previously been compared, we did not formulate a direct hypothesis on this matter.

Third, based on previous findings that individuals from collectivistic societies have lower levels of self-esteem [44–47], we hypothesized that individuals from East Germany would have lower self-esteem scores (assessed by the Rosenberg Self-Esteem Scale, RSE) compared with individuals from West Germany.

#### Main effect for age cohort

Learning environments can shape personality [74–76]; therefore we used trichotomized age cohorts to analyze narcissism and self-esteem. The youngest age cohort consisted of individuals ≤ 5 years of age in 1989. These individuals had not yet entered school in 1989. The middle age cohort included individuals aged between 6 (entered school in 1989) and 18 (the legal age of consent in 1989) years of age. The oldest age cohort contained individuals that were older than 18 years in 1989.

Individuals from the youngest age cohort were socialized within reunified Germany and shared a similar learning environment; therefore we did not expect to find any differences in grandiose narcissism, vulnerable narcissism, and self-esteem between individuals from East and West Germany in this cohort.

Based on the theory that learning environments shape personality [76], we hypothesized that differences in grandiose narcissism and self-esteem would be more pronounced in the middle age cohort, because these individuals were exposed to either individualistic or collectivistic conditions during a critical period of personality development. We expected that individuals from West Germany would have higher scores for grandiose narcissism and self-esteem compared with individuals from East Germany in this cohort.

We investigated whether grandiose narcissism, vulnerable narcissism, and self-esteem are more or less pronounced in individuals from East and West Germany in the oldest cohort. Two studies have shown that grandiose narcissism decreases [48, 50] and self-esteem increases [60, 61] in later life, suggesting that group differences may be less pronounced in the oldest cohort. However, narcissism and self-esteem have not been compered across age cohorts; therefore we did not formulate a direct hypothesis for the main effects.

## Materials and methods

### Participants and procedure

A total of 1,025 individuals (age in years: *M*_age_ = 38.3, *SD* = 12.48, range: 18–83; males 31.2%, females 68.8%) from Germany were recruited for this study by advertisements on a social networking site. Participants were invited to complete an online study and were given the opportunity to obtain an e-book as an incentive. All procedures were approved by the ethics committee of the Charité Berlin (EA04/065/06). All participants provided written informed consent by checking a box that stated they agreed to participate in the study; participants could only then proceed with the study. Afterwards, all participants had to insert their age, their level of education (i.e., no school degrees/German: keinen Schulabschluss; secondary modern school qualification 9^th^ class degree/German: Hauptschulabschluss; secondary modern school qualification 10^th^ class degree/German: Realschulabschluss; academic high school degree/German: Abitur; University degree/German: Hochschulabschluss) and town in which they grew up. A research assistant used an online map service to locate whether towns were part of former GDR or FRG.

Three hundred and forty-three participants grew up in the territory of the former GDR and 682 individuals grew up in the territory of the FRG before 1990. Regarding education, two individuals did not have a school degree, 82 had a secondary school degree, 177 had a graduation diploma, 595 had a university degree, and 164 received vocational training. In terms of employment, 72 participants were unemployed, 691 were employed, 223 were students, and 34 were retired. Five individuals did not provide information on their education and occupation.

Trichotomized groups were created based on the age of participants when the Berlin Wall fell in 1989. The youngest cohort (≤ 5 years of age in 1989) included 29.7% of the total participants, the middle age cohort (aged 6–18 years in 1989) contained 35.5% of the total participants, and the older cohort (aged 19–41 years in 1989) comprised 34.8% of the total participants.

### Measures

#### Grandiose narcissism (NPI)

We used a 15-item version of the NPI [77], which is the most widely used self-report measure of narcissism [7, 8, 78, 79]. The NPI primarily measures grandiose aspects of narcissism [13, 80], based on the criteria for pathological narcissism specified in the DSM III [15]. The coefficient alpha was 0.76.

#### Grandiose and vulnerable narcissism (PNI)

We used the German version of the PNI [13, 18], which contains 54 items for measuring grandiose and vulnerable narcissism. The PNI is comprised of seven subscales: exploitativeness (EXP, seven items), grandiose fantasy (GF, seven items), self-sacrificing self-enhancement (SSSE, six items), entitlement rage (ER, eight items), devaluing (DEV, seven items), contingent self-esteem (CSE, 12 items), and hiding the self (HS, seven items). Each item is scored on a six-point scale ranging from zero (not at all like me) to five (very much like me). The coefficient alpha was 0.96 for the total score, 0.71 for the grandiose factor (including the EXP, GF, ER and SSSE subscales [13] and 0.84 for the vulnerable factor (including the CSE, HS, and DEV subscales [13]. The alphas for the subscales ranged between 0.83 (SSSE) and 0.93 (CSE).

#### Self-esteem

The RSE [19, 79] is a 10-item self-report measure of global self-esteem. Items are rated on a 4-point scale ranging from 1 (strongly disagree) to 4 (strongly agree). The coefficient alpha in this sample was 0.90.

## Results

### Descriptive statistics

S1 Table presents the descriptive statistics and group differences between participants from East and West Germany across age classes. S2 Table shows the intercorrelations of all measures. There were no significant differences in gender and years of education between the East and West Germany groups. In both groups, the NPI scores correlated positively with PNI grandiosity scores, but negatively with PNI vulnerability scores. Self-esteem correlated positively with NPI narcissism and negatively with grandiosity and vulnerability assessed by the PNI. Using the Fisher r-to-z transformation, we calculated whether the correlations between NPI, PNI and RSE significantly differ between East and West Germany in each age class. There were no significant differences in correlations for all measures in each age class.

### Group differences

Participants who grew up in West Germany had lower self-esteem scores (RSE) and higher grandiose narcissism scores (NPI and PNI-G) compared with participants who grew up in East Germany (see S1 Table). Although the sum score of all facets of vulnerable narcissism did not differ between groups across age classes, participants from West Germany had by trend higher scores in the HS facet of vulnerable narcissism compared with individuals from East Germany. There were no significant differences in other PNI-V subscales between the two groups (i.e., CSE, and DEV).

In summary, individuals from West Germany scored higher on narcissistic grandiosity compared with participants from East Germany, but no differences in narcissistic vulnerability were observed. Participants who grew up in West Germany were significantly older than participants who grew up in East Germany. However, controlling for age and gender did not alter the results for differences in self-esteem, grandiose narcissism, and vulnerable narcissism. Levene's Test for Equality of Variances was statistically nonsignificant, which indicates that the group variances are equal in the population.

### Age cohort effect

To determine whether narcissism and self-esteem is different between individuals who grew up before or after the German unification, we performed a multivariate analysis of variance (MANOVA) according to age (young vs. middle vs. old) and group (East Germany vs. West Germany) (see S3 Table). These analyses revealed that grandiose narcissism, vulnerable narcissism, and self-esteem were different between age cohorts. Further, a group-effect was predominant in the middle age group; individuals who grew up in West Germany had higher grandiose narcissism and vulnerable narcissism scores and lower self-esteem scores compared with individuals in the same age group who grew up in East Germany. Individuals in the middle age group from West Germany had higher grandiose narcissism on three different PNI-G subscales (EXP, GF, and ER facets) as well as one PNI vulnerability subscale (HS facet) compared with participants from East Germany in the same age group (see S3 Table).

There were no significant effects in the youngest age cohort. Individuals in the oldest age group from West Germany had higher NPI scores compared with participants from East Germany in the same age group. The principal findings related to grandiose narcissism, vulnerable narcissism, and self-esteem in all three age classes are illustrated in S1 Fig. Controlling for age and gender does not alter the pattern of results.

## Discussion

This study takes advantage of the natural experiment [69, 70] created by the division and reunification of Germany to examine whether political systems affect narcissism and self-esteem. We compared measures of grandiose narcissism, vulnerable narcissism, and self-esteem between individuals who grew up in the territory of former West and East Germany. We hypothesized that individuals who grew up in the individualistic and capitalistic society of West Germany would have higher grandiose narcissism, and self-esteem scores than individuals who were raised in the collectivistic and socialist society of East Germany. By analyzing a large, heterogeneous community sample, we demonstrated that individuals from former West Germany have higher grandiose narcissism scores than individuals who grew up in former East Germany, in agreement with our hypothesis. In contrast to our hypothesis, individuals from former East Germany had higher self-esteem than individuals from former West Germany. These findings were predominantly detected in the middle age cohort and were largely absent in the youngest and oldest cohorts. In the following, we discuss our findings in relation to the proposed narcissism epidemic [1].

### Why were differences in narcissism predominant in the middle age cohort?

#### Youngest age cohort

Personality traits emerge during childhood and adolescence and become more stable in adulthood [81, 82]. Political systems may affect the educational system, therefore influence personality development [83]. Individuals from the youngest cohort (≤ 5 years old in 1989) grew up under a common political and cultural system in the capitalism-orientated society of reunified Germany. In line with this, we found no significant differences in any study measures between individuals who grew up in East or West Germany.

#### Middle age cohort

Participants in the middle age cohort (6–18 years old in 1989) grew up either in collectivistic East Germany or individualistic West Germany. Grandiose narcissism and vulnerable narcissism scores were higher in individuals from West Germany compared to East Germany in this age group.

Interestingly, individuals raised in West Germany scored higher on three grandiose subscales of the PNI: EXP (interpersonal manipulativeness), GF (compensatory fantasies of success and recognition), and ER (anger in response to unmet expectations that they feel entitled to).

Moreover, significant differences were observed in the HS subscale (vulnerable subscale of the PNI: concealing faults and needs from others), but not in the other two vulnerable subscales (CSE, DEV). These observations are in line with previous studies arguing that grandiose (but not necessarily vulnerable) narcissism has increased in Western societies [63]. Furthermore, our study sample may represent non-clinical individuals, which may explain why no significant differences were observed in two of the three vulnerable PNI subscales in the middle age cohort. There is previously published data showing that vulnerable aspects of the PNI are more strongly associated with psychopathology than grandiose facets [13, 84]. Moreover, grandiose aspects of the PNI are associated with tendencies to view oneself as active and energetic and one's life as exciting [84].

Increased grandiose narcissism in middle-aged individuals from West Germany may be explained by social learning theory [85]. Overvaluation of children by their parents has previously been hypothesized as the origin of narcissism in children in individualistic societies [85]. In a prospective study, Brummelman and colleagues confirmed preliminary data [86] that parental overvaluation predicted narcissism in the child later in life [87]. It remains to be confirmed whether differences in parental overvaluation existed between the former East and West Germany.

Moreover, the work by Bianchi argues that narcissistic self-focus may be viable during economic prosperous times and tempered by economic recession [54]. Individuals from GDR who came of age during the reunion of Germany faced tumultuous economic times including the loss of job perspectives (e.g., break down of textile industry and agriculture). Moreover, East German wages and pensions continue to be below the West German wage level since unification [88]. Middle-aged individuals from East Germany may score lower on narcissism as they experience lower economic prosperity compared to middle-aged individuals from West Germany.

Finally, data from an internet sample from China assessing sociodemographic factors related to grandiose narcissism (NPI) might help to explain differences in the middle age cohort in our study [48]. The authors found higher socioeconomic status, offspring of one child families compared to multiple children, living in urban area compared to rural area positively related to grandiose narcissism [48]. Up from the mid-seventies East Germany had higher birth-rates compared to West-Germany [89]. Lower socio-economic status in East-Germany compared to West-Germany has already been described and continues to exist [88]. Furthermore, urbanization in West-Germany was much faster between 1949 and 1989/90 compared to East-Germany [90].

#### Oldest age cohort

Grandiose narcissism measured with the PNI-G was higher in participants who grew up in West Germany than East Germany in the oldest age cohort, but no significant differences were detected in grandiose narcissism measured with the NPI. Prior data has shown that grandiose narcissism measured with the NPI is widely captured by the exploitative (EXP) subscale of the PNI grandiosity scales which reflects a manipulative interpersonal orientation [91]. In the current study, the exploitative PNI subscale had highest correlation with the NPI score and was by trend higher in the oldest age cohort who grew up in West Germany than East Germany. This discrepancy in grandiose narcissism between the middle and oldest age group may be because narcissism decreases with age [92]. Older individuals have lower narcissism scores [49, 50] and are less susceptible to personality disorders than younger subjects [93]. Individuals from the oldest age group were in their fifties at the time of the study and may have felt an increased sense of empowerment, security, personal growth, and success than younger participants [94]. NPI-assessed grandiose narcissism may be more resistant to change throughout life because of its beneficial aspects. Furthermore, some of the mentioned factors valid for the middle age cohort might be less relevant or not applicable for the oldest age cohort, e.g., differences in birth rate, urbanization, tumultuous economic times during adolescents and early adulthood.

### Why was self-esteem higher in participants from East Germany?

Our finding that self-esteem was higher in individuals from East Germany contradicts previous reports that individuals from more collectivistic East Asian countries have lower self-esteem than individuals from Western cultures [43–47]. We conclude that these earlier findings may not apply to East and West Germany. Furthermore, the assumption that individuals from collectivistic East Asian countries have lower self-esteem than individuals from Western cultures has been stressed by theoretical considerations and empirical data. Sedikides and colleagues argue that one cannot conclude that the level of self-esteem is weaker in East Asian individuals compared to Western individuals [95]. They argue that the desire for self-esteem is pancultural (i.e., cross-cultural invariance), with differential manifestations of self-esteem in different cultures (i.e., cross-cultural variability). They propose that more refined theoretical formulations and self-report instruments with a high cross cultural sensitivity are needed to better understand within-culture variations in self-esteem (including differences between East-Asian cultures, Latino and Middle-Eastern cultures and other Eastern countries).

Furthermore, teaching children individualistic virtues may contribute to lower self-esteem. According to Tafarodi and Walters, individualistic societies promote achievement-dependent self-esteem [96], i.e., a self-esteem that is threatened by constant social comparisons and the necessity to achieve more than other individuals. In line with this, narcissists perform better when self-enhancement opportunity is high rather than low, while the performance of participants with low narcissism is relatively unaffected by self-enhancement opportunity [97]. Individuals who grew up in West Germany may have lower, achievement-dependent self-esteem (i.e., which may be unrelated to actual achievement). In contrast, collectivistic societies are directed toward maintaining social harmony [42]. Individuals who grew up in East Germany may experience higher self-esteem, because it appears to be more independent from achievements and social comparisons.

Finally, Brummelman and colleagues demonstrated that parental warmth reported by the child was predicative of the child’s self-esteem, whereas parent-reported parental warmth was not [87]. It is possible that child-perceived parental warmth was more prominent in former East Germany compared with former West Germany.

### Strengths and limitations

The strengths of this study are the large sample size and the combined assessment of grandiose narcissism, vulnerable narcissism, and self-esteem. Most importantly, this study takes advantage of the natural experiment [69, 70] created by the German division and reunification. In this setting, participants share important characteristics (such as language), but are distinct in others (such as sociocultural education). Our findings have advanced our understanding of the sociocultural mechanisms underlying individual differences in narcissism.

However, there are some methodological limitations to our study that could be addressed by future research. First, our study is based on associations, and conclusions about causal effects should be tested using longitudinal designs. Second, our methods for assessing narcissism and self-esteem were based on self-reports, which have limited value for assessing personality [98]. In future studies, negative behaviors associated with narcissism in real life situations should be compared between individuals raised in the former East and West Germany [99–101]. Third, we cannot determine whether our participants answered the online questionnaire honestly. This criticism also applies to offline questionnaires; therefore peer reports or naturalistic observation methods might represent a better approach towards investigating the interpersonal behavior of narcissists [102, 103]. Future studies may use paper pencil tests and more conventional sampling methods. Fourth, we cannot exclude confounding differences between the East German and West German cohorts. Individuals who grew up in the former East Germany experienced a transition from a collectivistic communist society to an individualistic society, which may have affected their narcissism and self-esteem. Moderating variables, such as differences in parenting styles should be investigated in future studies [87].

## Conclusions

In summary, our study provides preliminary evidence that sociocultural factors contribute to differences in narcissism. Although we cannot pinpoint any causal relationships, we believe that our study sheds further light on sociological trends and justifies further investigation into the alleged narcissism epidemic.

## Supporting information

S1 Table Descriptive statistics and MANCOVA effect for group differences between East and West Germany across age classes (controlled for age). NPI = Narcissistic Personality Inventory; PNI = Pathological Narcissism Inventory; PNI-G/-V = Pathological Narcissism Inventory grandiose/vulnerable narcissism (according to Pincus et al. 2009); EXP = Exploitativeness; SSSE = Self-Sacrificing Self-Enhancement; HS = Hiding the Self; GF = Grandiose Fantasy; DEV = Devaluing; ER = Entitlement Rage; RSE = Rosenberg Self-Esteem Scale; only RSE, PNI and NPI Scales are controlled for age; d = effect size Cohens d.
(DOCX)

S2 Table Correlations of all variables. N = 1,025; intercorrelations for individuals from former East Germany above diagonal (N = 343), intercorrelations for individuals from former West Germany below diagonal (N = 682); NPI = Narcissistic Personality Inventory; PNI = Pathological Narcissism Inventory; PNI-G/-V = Pathological Narcissism Inventory grandiose/vulnerable narcissism; EXP = Exploitativeness; SSSE = Self-Sacrificing Self-Enhancement; HS = Hiding the Self; GF = Grandiose Fantasy; DEV = Devaluing; ER = Entitlement Rage; RSE = Rosenberg Self-Esteem Scale; * p < .05, ** p < .01, *** p < .001.
(DOCX)

S3 Table Descriptive statistics and MANOVA effect for group differences between East and West Germany across age classes. NPI = Narcissistic Personality Inventory; PNI = Pathological Narcissism Inventory; PNI-G/-V = Pathological Narcissism Inventory grandiose/vulnerable narcissism; EXP = Exploitativeness; SSSE = Self-Sacrificing Self-Enhancement; HS = Hiding the Self; GF = Grandiose Fantasy; DEV = Devaluing; ER = Entitlement Rage; RSE = Rosenberg Self-Esteem Scale; d = effect size Cohens d.
(DOCX)

S1 Fig **(1a) Age cohort effect for self-esteem (RSE). (1b) Age cohort effect for narcissism (NPI). (1c) Age cohort effect for grandiose narcissism (PNI-G). (1d) Age cohort effect for vulnerable narcissism (PNI-V).** NPI = Narcissistic Personality Inventory; PNI = Pathological Narcissism Inventory; PNI-G/-V = Pathological Narcissism Inventory grandiose/vulnerable narcissism; * p < .05, ** p < .01.
(TIF)

S1 File Ethics statement. (PDF)
