# Correction: Does a narcissism epidemic exist in modern western societies? Comparing narcissism and self-esteem in East and West Germany

**DOI:** 10.1371/journal.pone.0198386

**Published:** 2018-05-29

**Authors:** Aline Vater, Steffen Moritz, Stefan Roepke

There is an error in the penultimate sentence of the Grandiose and vulnerable narcissism (PNI) section. The correct sentence is: Each item is scored on a six-point scale ranging from zero (not at all like me) to six (very much like me). The coefficient alpha was 0.96 for the total score, 0.71 for the grandiose factor (including the EXP, GF, ER and SSSE subscales [13] and 0.84 for the vulnerable factor (including the CSE, HS, and DEV subscales [13].

In S1 Fig, part 1c is labelled as 1b. Please see the corrected S1 Fig here.

## Supporting information

S1 Fig **(1a) Age cohort effect for self-esteem (RSE). (1b) Age cohort effect for narcissism (NPI). (1c) Age cohort effect for grandiose narcissism (PNI-G). (1d) Age cohort effect for vulnerable narcissism (PNI-V)**. NPI = Narcissistic Personality Inventory; PNI = Pathological Narcissism Inventory; PNI-G/-V = Pathological Narcissism Inventory grandiose/vulnerable narcissism; * p < .05, ** p < .01.
(TIF)
