# Supplementary material for: Does a narcissism epidemic exist in modern western societies? Comparing narcissism and self-esteem in East and West Germany
Source: PLoS One. 2018 Jan 24;13(1):e0188287. doi: 10.1371/journal.pone.0188287 (PMC5783345; doi:10.1371/journal.pone.0188287)
Supplement: S1 Table — NPI = Narcissistic Personality Inventory; PNI = Pathological Narcissism Inventory; PNI-G/-V = Pathological Narcissism Inventory grandiose/vulnerable narcissism (according to Pincus et al. 2009); EXP = Exploitativeness; SSSE = Self-Sacrificing Self-Enhancement; HS = Hiding the Self; GF = Grandiose Fantasy; DEV = Devaluing; ER = Entitlement Rage; RSE = Rosenberg Self-Esteem Scale; only RSE, PNI and NPI Scales are controlled for age; d = effect size Cohens d. (DOCX) [file pone.0188287.s001.docx]

S1 Table.

|  | **East Germany (n=343)** | | **West Germany (n=682)** | |  | | |  |  |
| --- | --- | --- | --- | --- | --- | --- | --- | --- | --- |
|  | *N* |  | *N* |  | ***χ^2^*** *Value* | *p* |  |  |  |
| **Gender** |  |  |  |  | 1.024 | .312 |  |  |  |
| Female | 243 |  | 462 |  |  |  |  |  |  |
| Male | 100 |  | 220 |  |  |  |  |  |  |
|  | *M* | *SD* | *M* | *SD* | *F* | *p* |  | *d* |  |
| **Age** | 37.00 | 11.34 | 38.78 | 12.57 | 4.88 | .027 |  | .14 |  |
| **Education** | 14.91 | 2.61 | 14.85 | 2.68 | .10 | .749 |  | .02 |  |
|  | *M* | *SD* | *M* | *SD* | *F* | *p* | *d* | |  |
| **NPI** | 4.40 | 2.92 | 5.19 | 3.27 | 10.13 | .002 |  | 0.25 |  |
| **PNI Total** | 3.01 | .77 | 3.10 | .78 | 5.51 | .019 |  | 0.12 |  |
| **PNI-G** | 3.09 | .73 | 3.20 | .76 | 6.90 | .009 |  | 0.15 |  |
| EXP | 3.01 | .90 | 3.19 | .96 | 7.25 | .007 |  | 0.19 |  |
| GF | 3.43 | 1.05 | 3.42 | 1.06 | 5.73 | .017 |  | 0.01 |  |
| ER | 3.13 | 1.06 | 3.27 | 1.10 | 5.01 | .025 |  | 0.13 |  |
| SSSE | 2.79 | .97 | 2.91 | 1.01 | .13 | .722 |  | 0.12 |  |
| **PNI-V** | 2.94 | .93 | 3.00 | .94 | 2.47 | .116 |  | 0.06 |  |
| CSE | 2.96 | 1.08 | 3.00 | 1.10 | 1.83 | .177 |  | 0.04 |  |
| HS | 3.17 | 1.08 | 3.29 | 1.08 | 3.77 | .052 |  | 0.11 |  |
| DEV | 2.69 | 1.06 | 2.72 | 1.09 | .59 | .442 |  | 0.03 |  |
| **RSE** | 32.33 | 5.56 | 31.74 | 6.00 | 4.05 | .044 |  | 0.10 |  |
