# Supplementary material for: Does a narcissism epidemic exist in modern western societies? Comparing narcissism and self-esteem in East and West Germany
Source: PLoS One. 2018 Jan 24;13(1):e0188287. doi: 10.1371/journal.pone.0188287 (PMC5783345; doi:10.1371/journal.pone.0188287)
Supplement: S2 Table — N = 1,025; intercorrelations for individuals from former East Germany above diagonal (N = 343), intercorrelations for individuals from former West Germany below diagonal (N = 682); NPI = Narcissistic Personality Inventory; PNI = Pathological Narcissism Inventory; PNI-G/-V = Pathological Narcissism Inventory grandiose/vulnerable narcissism; EXP = Exploitativeness; SSSE = Self-Sacrificing Self-Enhancement; HS = Hiding the Self; GF = Grandiose Fantasy; DEV = Devaluing; ER = Entitlement Rage; RSE = Rosenberg Self-Esteem Scale; * p < .05, ** p < .01, *** p < .001. (DOCX) [file pone.0188287.s002.docx]

S2 Table.

|  |  | 1 | 2 | 3 | 4 | 5 | 6 | 7 | 8 | 9 | 10 | 11 | 12 |
| --- | --- | --- | --- | --- | --- | --- | --- | --- | --- | --- | --- | --- | --- |
| 1 | **NPI** |  | -.03 | .20*** | .48*** | .13* | .00 | .02 | -.18** | -.15** | -.14* | -.17** | .34*** |
| 2 | **PNI Total** | .04 |  | .86*** | .24*** | .76*** | .82*** | .67*** | .93*** | .88*** | .75*** | .80*** | -.53*** |
| 3 | **PNI-G** | .30*** | .86*** |  | .53*** | .83*** | .77*** | .78*** | .63*** | .61*** | .49*** | .54*** | -.27** |
| 4 | EXP | .54*** | .30*** | .57*** |  | .23*** | .21*** | .18** | .03 | -.02 | .06 | .04 | .19** |
| 5 | GF | .22*** | .74*** | .81*** | .27*** |  | .57*** | .57*** | .58*** | .57*** | .47*** | .48*** | -.31*** |
| 6 | ER | .10*** | .80*** | .78*** | .24*** | .55*** |  | .47*** | .69*** | .70*** | .46*** | .64*** | -.39*** |
| 7 | SSSE | .04 | .64*** | .76*** | .21*** | .51*** | .47*** |  | .50*** | .49*** | .42*** | .40*** | -.24*** |
| 8 | **PNI-V** | -.15*** | .91*** | .58*** | .08 | .53*** | .63*** | .44*** |  | .89*** | .84*** | .88*** | -.61*** |
| 9 | CSE | -.11*** | .89*** | .63*** | .08 | .59*** | .66*** | .50*** | .88*** |  | .60*** | .72*** | -.64*** |
| 10 | HS | -.13*** | .71*** | .40*** | .05 | .36*** | .44*** | .30*** | .85*** | .60*** |  | .59*** | -.44*** |
| 11 | DEV | -.16*** | .77*** | .48*** | .07 | .42*** | .52*** | .36*** | .88*** | .68*** | .60*** |  | -.50*** |
| 12 | **RSE** | .37*** | -.49*** | -.17*** | .21*** | -.26^**^ | -.28^**^ | -.14^**^ | -.62*** | -.61*** | -.46*** | -.54*** |  |
