# Supplementary material for: Does a narcissism epidemic exist in modern western societies? Comparing narcissism and self-esteem in East and West Germany
Source: PLoS One. 2018 Jan 24;13(1):e0188287. doi: 10.1371/journal.pone.0188287 (PMC5783345; doi:10.1371/journal.pone.0188287)
Supplement: S3 Table — NPI = Narcissistic Personality Inventory; PNI = Pathological Narcissism Inventory; PNI-G/-V = Pathological Narcissism Inventory grandiose/vulnerable narcissism; EXP = Exploitativeness; SSSE = Self-Sacrificing Self-Enhancement; HS = Hiding the Self; GF = Grandiose Fantasy; DEV = Devaluing; ER = Entitlement Rage; RSE = Rosenberg Self-Esteem Scale; d = effect size Cohens d. (DOCX) [file pone.0188287.s003.docx]

S3 Table.

|  |  |  | Descriptive statistics | | | | | | | | | | | | | |  | Comparisons between age classes | | | | |  | Comparisons within age classes | | | | | | | | | | | | | | | | |
| --- | --- | --- | --- | --- | --- | --- | --- | --- | --- | --- | --- | --- | --- | --- | --- | --- | --- | --- | --- | --- | --- | --- | --- | --- | --- | --- | --- | --- | --- | --- | --- | --- | --- | --- | --- | --- | --- | --- | --- | --- |
|  | Age **≤** 5 | | | | | Age 6 to 18 | | | | | | Age **≥** 19 | | | | |  |  | | |  | |  | Age **≤** 5 | | | | Age 6 to 18 | | | | | | | Age **≥** 19 | | | | | |
|  | East (N = 105) | | West (N = 192) | |  | | East (N = 133) | | West (N = 236) | |  | | East (N = 105) | | West (N = 254) | |  | Age class | | East-West | | |  | East-West | | | | East-West | | | | | | | East-West | | | | | |
|  | *M* | *SD* | *M* | *SD* |  | | *M* | *SD* | *M* | *SD* |  | | *M* | *SD* | *M* | *SD* |  | *F* | *p* | *F* | | *p* |  | *F* | *p* | *d* | | *F* | *p* | | | *d* | | *F* | | *p* | | *d* | | |
| **NPI** | 4.32 | 3.04 | 4.58 | 3.04 |  | | 4.27 | 2.88 | 5.17 | 3.13 |  | | 4.67 | 2.87 | 5.70 | 3.51 |  | 3.24 | .040 | 9.82 | | .002 |  | .15 | .699 | .09 | 7.97 | | | .005 | .30 | | 5.15 | | | | .024 | | .32 |  |
| **PNI** | 3.25 | .70 | 3.27 | 0.72 |  | | 2.99 | .75 | 3.24 | .73 |  | | 2.78 | .80 | 2.83 | .79 |  | 24.01 | .000 | 4.13 | | .043 |  | .05 | .823 | .03 | 8.69 | | | .003 | .34 | | .29 | | | | .593 | | .06 |  |
| **PNI-G** | 3.33 | .66 | 3.31 | 0.71 |  | | 3.08 | .71 | 3.34 | .70 |  | | 2.86 | .75 | 2.99 | .80 |  | 20.18 | .000 | 5.45 | | .020 |  | .09 | .755 | .03 | 10.26 | | | .001 | .37 | | 1.76 | | | | .185 | | .17 |  |
| EXP | 3.09 | .89 | 3.14 | 0.97 |  | | 2.99 | .87 | 3.24 | .94 |  | | 2.96 | .96 | 3.18 | .98 |  | .21 | .814 | 6.72 | | .010 |  | .16 | .688 | .05 | 6.06 | | | .014 | .28 | | 3.04 | | | | .082 | | .23 |  |
| GF | 3.43 | .91 | 3.42 | 1.05 |  | | 3.11 | 1.07 | 3.47 | 1.03 |  | | 2.85 | 1.10 | 2.98 | 1.14 |  | 15.86 | .000 | 4.60 | | .032 |  | .00 | .957 | .01 | 8.91 | | | .003 | .34 | | .86 | | | | .355 | | .12 |  |
| ER | 2.94 | .92 | 3.02 | 0.92 |  | | 2.84 | 1.02 | 3.13 | .98 |  | | 2.56 | .93 | 2.63 | 1.06 |  | 13.67 | .000 | 4.20 | | .041 |  | .36 | .547 | .09 | 6.50 | | | .011 | .29 | | .27 | | | | .607 | | .07 |  |
| SSSE | 3.87 | 1.02 | 3.65 | 1.04 |  | | 3.37 | .98 | 3.50 | .97 |  | | 3.05 | 1.01 | 3.16 | 1.11 |  | 24.99 | .000 | .01 | | .943 |  | 2.88 | .091 | .21 | 1.30 | | | .256 | .13 | | .66 | | | | .416 | | .10 |  |
| **PNI**-V | 3.17 | .89 | 3.20 | 0.90 |  | | 2.90 | .93 | 3.15 | .90 |  | | 2.74 | .93 | 2.72 | .95 |  | 15.90 | .000 | 1.68 | | .195 |  | .06 | .814 | .03 | 5.32 | | | .022 | .27 | | .02 | | | | .890 | | .02 |  |
| CSE | 3.27 | 1.07 | 3.34 | 1.04 |  | | 2.92 | 1.02 | 3.14 | 1.04 |  | | 2.67 | 1.08 | 2.62 | 1.09 |  | 24.52 | .000 | 1.15 | | .283 |  | .30 | .587 | .07 | 3.42 | | | .065 | .21 | | .14 | | | | .707 | | .05 |  |
| HS | 3.42 | 1.12 | 3.34 | 1.01 |  | | 3.11 | 1.08 | 3.47 | 1.06 |  | | 2.96 | 1.00 | 3.07 | 1.11 |  | 8.12 | .000 | 2.91 | | .089 |  | .35 | .554 | .08 | 8.35 | | | .004 | .34 | | .65 | | | | .420 | | .10 |  |
| DEV | 2.82 | 1.11 | 2.91 | 1.11 |  | | 2.68 | 1.03 | 2.83 | 1.06 |  | | 2.58 | 1.04 | 2.47 | 1.05 |  | 6.68 | .001 | .34 | | .560 |  | .38 | .537 | .08 | 1.57 | | | .211 | .14 | | .67 | | | | .415 | | 11 |  |
| **RSE** | 30.35 | 6.00 | 30.29 | 5.9 |  | | 32.85 | 5.28 | 31.11 | 5.99 |  | | 33.66 | 4.93 | 33.41 | 5.70 |  | 19.29 | .000 | 2.85 | | .092 |  | .04 | .845 | .01 | 7.14 | | | .008 | .31 | | .04 | | | | .837 | | .05 |  |
