# Supplementary figures and images for: Does a narcissism epidemic exist in modern western societies? Comparing narcissism and self-esteem in East and West Germany
Source: PLoS One. 2018 Jan 24;13(1):e0188287. doi: 10.1371/journal.pone.0188287 (PMC5783345; doi:10.1371/journal.pone.0188287)

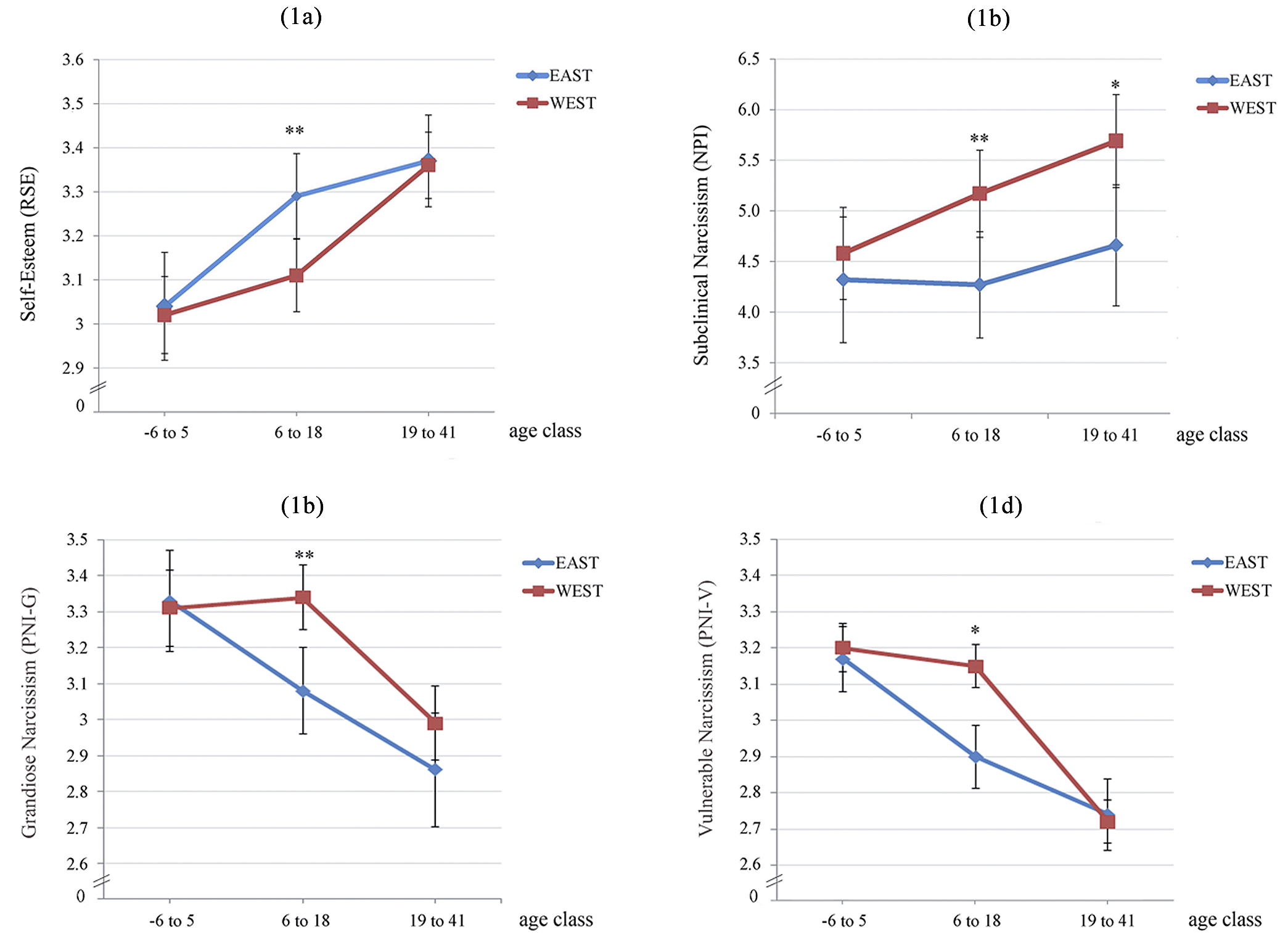

Supplement: S1 Fig — (1a) Age cohort effect for self-esteem (RSE). (1b) Age cohort effect for narcissism (NPI). (1c) Age cohort effect for grandiose narcissism (PNI-G). (1d) Age cohort effect for vulnerable narcissism (PNI-V). NPI = Narcissistic Personality Inventory; PNI = Pathological Narcissism Inventory; PNI-G/-V = Pathological Narcissism Inventory grandiose/vulnerable narcissism; * p < .05, ** p < .01. (TIF) [file pone.0188287.s004.tif]
