# Supplementary material for: Does a narcissism epidemic exist in modern western societies? Comparing narcissism and self-esteem in East and West Germany
Source: PLoS One. 2018 Jan 24;13(1):e0188287. doi: 10.1371/journal.pone.0188287 (PMC5783345; doi:10.1371/journal.pone.0188287)
Supplement: S1 File — (PDF) [file pone.0188287.s005.pdf]

CHARITÉ

Charité | 10117 Berlin

Herrn
Dr. med. Stefan Röpke
Klinik und Hochschulambulanz für Psychiatrie
und Psychotherapie

CBF

Ethikkommission
Ethikausschuss 4 am Campus Benjamin Franklin
Vorsitzender: Prof. Dr. Ralf Stahlmann

Geschäftsführung: Katja Orzechowski
ethikkommission@charite.de

Korrespondenzadresse: Charitéplatz 1, 10117 Berlin
Tel.: 030/450-517222
Fax: 030/450-517952
www.charite.de/ethikkommission

Datum: 12.01.11

Psychometrische Charakterisierung von Patienten mit Persönlichkeitsstörungen der Cluster A, B oder C nach DSM IV (Paranoide-, Schizoide-, Schizotypische-, Antisoziale-, Borderline-, Histrionische-, Narzisstische-, Vermeidend-Selbstunsichere-, Dependente-, zwanghafte und nicht näher bezeichnete Persönlichkeitsstörung) und Erfassung von Persönlichkeitsmerkmalen (u.a. Fünf Faktoren Modell, Empathiefähigkeit, Theory of Mind, Ängstlichkeit, Depressivität)

Antragsnummer: EA4/065/06
Vorgang vom 27.12.10, Eingang am 29.12.10

Sehr geehrter Herr Dr. Röpke,

hiermit bestätigen wir Ihnen den Eingang Ihres Schreibens vom 27.12.10, in dem Sie eine zweite Studienänderung beantragten und folgende Anlagen beifügten:

- Patienteninformation vom 27.12.10
- Probandeninformation vom 27.12.10
- Einwilligungserklärung vom 27.12.10
- Fragebögen

Wir danken für die Kenntnisgabe. Die Ethikkommission erhebt keine Einwände gegenüber den Änderungen.

Mit freundlichen Grüßen

R. Stahlmann

Prof. Dr. R. Stahlmann
-Vorsitzender-
